# Patterns of a culture of aggression amongst Grade 10 learners in a secondary school in the Sedibeng District, South Africa

**DOI:** 10.4102/curationis.v38i1.1233

**Published:** 2015-03-27

**Authors:** Chris Myburgh, Marie Poggenpoel, Lovia Nhlapo

**Affiliations:** 1 Department of Educational Psychology, University of Johannesburg, South Africa; 2 Department of Nursing Science, University of Johannesburg, South Africa

## Abstract

**Background:**

A number of reports to the Department of Education indicated high levels of aggression in a Grade 10 A class in a secondary school in Sedibeng District, Gauteng. Teachers, the school management team, school governing body, school-based support team, parents, community leaders and learners seemed unable to manage this constructively. Neither the culture of aggression nor the influence of this phenomenon on those entrapped in it were understood. No published research reports could be found on cultures of aggression in South African secondary schools. There was therefore a dire need to explore and describe the culture of aggression in this specific Grade 10 A class.

**Objectives:**

This article reports on patterns of a culture of aggression observed amongst learners in a Grade 10 class in a secondary school in the Sedibeng District of the Gauteng Department of Education.

**Method:**

A qualitative, exploratory, descriptive and contextual research design was followed with an ethnographic approach. Purposive sampling was used to select participants. Data consisted of observations of ‘rich points’, interviews and field notes, and thematic data analysis and an independent coder were used.

**Results:**

Findings reflected four patterns of a culture of aggression amongst learners, namely patterns of anger, bullying, fighting, and challenges to moral values. At the root of these were neglect of and non-adherence to human rights and a sound base of morals.

**Conclusion:**

The challenge is to assist the involved learners to respect each other's human dignity, so that relationships can be developed in which those involved act with sensitivity towards each other's needs. Such relationships often also result in the development of self-respect and a nuanced future orientation as part and parcel of mental health.

## Introduction

Allen (2009:103) asserts that aggression is a social act and that various forms of socially acceptable aggression are reinforced on a daily basis through the media, public discourse and teaching. In line with this, Grossman (2010:309) states that society inadvertently desensitises vulnerable learners to aggression and socialises them to accept it as a means of coping with their social environment and life's challenges. He believes that this is a cultural shift that accounts for much of the tragic learner aggression occurring in schools.

As a microcosm of society, secondary schools are not immune to the aggression portrayed by society. Marie Poggenpoel and Chris Myburgh (2008:3) of the University of Johannesburg said that such aggression in South African schools was a reflection of what was happening in society. Professor Poggenpoel made this comment following a sword attack on learners and staff at a secondary school in Krugersdorp. Various explanations were offered as to why South African learners are disproportionately involved in aggression and crime; some of these relate to the vestiges of South Africa's past and to the high level of political and other forms of aggression to which learners are exposed.

The world report on aggression and health (World Health Organization 2002) outlines risk factors for learners’ aggression at different levels, that impacts on secondary schools and society at large. The different levels of aggression include, for example, the individual level, relationship level and community level. The individual level focuses on self-esteem and a history of early aggressive behaviour. The relationship level focuses on teachers’ poor supervision of learners, combined with parents’ often harsh physical punishment of learners. The community level focuses on the socio-economic status of families as well as various cultural and societal levels, for example, the presence of gangs, guns and drugs in the community, low levels of social cohesion, income inequality, poor law enforcement and the normative use of violence to resolve conflict.

A culture of aggression is a matter of concern in secondary schools in South Africa today. According to Botha, Myburgh and Poggenpoel (2012:412–414), a culture of aggression possesses the potential to harm learners’ daily interaction with peers, as well as the school community at large. It may also have the potential to affect learners’ mental health negatively.

The researchers have observed countless instances of a culture of aggression in various parts of the secondary school environment, for example in the classroom, school assembly, school corridors, the school tuckshop, on school playgrounds and in school toilets. Dake et al. (2003:173–180) assert that the secondary school is without a doubt a place where high levels of aggression occur.

Schools are more and more being seen as less benign and safe (Dabbs & Leventhal 1966:525–531). According to the previous authors, aggression in a secondary school is often expressed visibly, audibly and by acts of violent behaviour conveying fear. A hotspot for the manifestation of a culture of aggression was observed by the researcher conducting fieldwork in secondary schools in the areas next to the secondary school yard and often when learners were walking to and from school. This culture of aggression was also observed in the school culture, for example related to initiation rituals, social class and gangs.

The nature of the organisational culture of a secondary school cannot be overlooked when exploring and describing a culture of aggression (Plummer 2001:15–23). In this context, Finnan and Levin (in Altrichter & Elliot 2000:23) emphasise the importance of acknowledging the secondary school's organisational culture explicitly, as it may have profound effects on a culture of aggression in a secondary school environment.

The constructive management of a culture of aggression has to take place at deeper levels of the secondary school as an organisation. It is imperative to examine the shared meaning attached to patterns of aggression by learners regarding their classroom and other places in the secondary school, such as the playgrounds, tuckshop and assembly. The shared meaning of learners may be reflected by their moral values and attitudes, to which they give meaning in their daily interactions in the school community (Davidhoff & Lazarus 2000:26).

Most present-day school aggression incidents happen in the form of verbal and non-verbal abuse, gang activities, disrespect, bullying and disorderly behaviour (Gilbert 2010:65). A phenomenon primarily occurring in secondary schools is the presence of weapons, such as sharp objects like self-made knives, guns, pieces of broken bottles, blades and scissors (Gilbert 2010:65). The use of weapons in schools became eminent when a North West Province learner stabbed another learner with a pair of scissors (Anderson & Bushman 2009:201). The Rosettenville secondary school shooting also demonstrated the extent of a culture of aggression in South Africa's secondary schools (South African Broadcasting Corporation 2006).

Many other incidences of violence related to aggression are reported by the media on a day-to-day basis. Banks (2003:10) states that learners find themselves without structured activities that are meant to absorb their energy as well as give them direction and a sense of self-worth in the school environment. According to Berry et al. (2002:214), learners who are not exposed to meaningful school activities spend a lot of time drifting on the school premises, until they come into contact with youth gangs.

A number of learners were left destitute in their classrooms, without supervision and tuition owing to the number of teachers who have been redeployed (Pena 2009:211). These imbalances, that the researchers also observed, are a result of the teacher-learner ratio and are some of the repercussions of teacher redeployment. The disintegration of a culture of learning, teaching and service resulted in a number of idling learners (Asmal 2005:321). A learner's idling mind can often be the breeding ground for a culture of aggression and aggressive behaviours.

The disintegration of a culture of learning, teaching and service is a recipe for lawlessness. According to Kassin, Fein and Markus (2008:56) lawlessness can promote a culture of aggression that leads to violence and other forms of behavioural problems in the classroom environment. A culture of aggression can give rise to violence, bullying, theft, truancy and gangsterism, feelings of fear, anxiety, retaliation, disrespect and lack of self-respect. Learners may leave school prematurely and show a lack of interest in schooling activities and a decline in academic achievements. It is as a result of this lack of interest in schooling activities and the decline in academic achievements that MacDonald (2004:27) is convinced that learners in schools are exposed to aggression (Breet, Myburgh & Poggenpoel 2010:515).

The accountability and commitment of the learners, teachers, parents and entities such as the school management team, school governing body, community, Department of Education (DoE), as well as other interdepartmental stakeholders, are important when incidents of aggression occur (Nhlapo 2013). Unfortunately these entities seemed to be in denial about the existence of a culture of aggression in the classroom (Pellegrini 2004:121).

## Problem statement

One of the authors is an education specialist in the DoE, whose duties include monitoring aggression in schools. A great number of reports to the DoE concerning aggressive incidents in the class and school indicated extremely high levels of aggression prevalent in a specific Grade 10 A class in a secondary school in the Sedibeng District, Gauteng. So far the teachers, school management team, school governing body, school-based support team, parents, community leaders and learners seemed to be unable to manage the culture of aggression in the Grade 10 class constructively. Neither the culture of aggression amongst learners in this Grade 10 class nor the influence this phenomenon had on the persons entrapped in the culture of aggression were understood. No published research reports could be found on cultures of aggression in South African secondary schools. There was therefore a dire need to explore and describe the culture of aggression that was present in this specific Grade 10 A class.

The research question that arose was: What is the culture of aggression amongst learners in a Grade 10 class in a secondary school in the Sedibeng District of the Gauteng DoE?

## Objective of this research

The research was guided by the following objective: to explore and describe the culture of aggression amongst learners in a Grade 10 class in a secondary school in the Sedibeng District of the Gauteng DoE.

## Research method and design

### Research design

In this research a qualitative, exploratory, descriptive and contextual research design was applied (Creswell 2007:35-47). A qualitative design was chosen to explore the culture of aggression, as a qualitative research method is largely an investigative process where the researchers make sense of a social phenomenon by identifying the patterns in it (Miles & Huberman 2009:104).

An exploratory research design was followed in order to explore the culture of aggression amongst learners in a Grade 10 class, as very little is known about the culture of aggression (Creswell 2007:35-47). This design would assist in understanding the culture of aggression in the context under study. The collected information on the culture of aggression from the participants’ perspective was explored and described (Brink & Wood 2006:119).

The aim was to describe the culture of aggression amongst learners rather than explaining it. The intention of the chosen design was to give an in-depth (Babbie & Mouton 2001:279–280) clarification of the specific situation of individuals and a group of people in an organisation to identify a culture of aggression. This research design was also contextual, in that the research was conducted in the Grade 10 A class in a secondary school in the Sedibeng District of the Gauteng DoE.

### Research method

An ethnographic approach was used to identify ‘rich points’ (Agar 1998:3–5) as well as ‘insider’ informants (Denzin & Lincoln 2006:652–656; Moustakis 1994:1–4; Creswell 2003:199–205). A rich point (Agar 1998:3–5) was distinguished as an indicator or index noticed in the observations and other collected data that was unusual and thought-provoking. These rich points constituted our experiences of that which needed clarification with regard to the culture of aggression. The rich points were identified before interaction with the participants, by means of interviews in order to build up an interrelated pattern of what exactly constituted the culture of aggression for learners in the Grade 10 A class.

Long-term engagements took place in the context of the classroom, school and even the environment. All these efforts were continuously scrutinised and studied to identify rich points. Trust was built between the interviewing researcher and prospective ‘insider’ participants of the culture of aggression. After data saturation had been obtained, this process stopped.

### Population and sample

A secondary school in Sedibeng District was identified by the district office of the Gauteng DoE as having a history of learners exhibiting aggressive behaviour. This secondary school is situated next to an informal housing settlement. The school-based support team coordinates all of the cases related to aggression that were referred by the schools to the district office on a monthly basis for analysis (DoE 2008:94). The criterion for inclusion in the sample was that the learners in Grade 10 were reported by the school-based support team to the district office of the Gauteng DoE as the most aggressive group of learners in a secondary school in the district. Class 10 A was selected to be in the sample.

In this study the purposive sample (Burns & Grove 2009:355) consisted of participants that included the Grade 10 A learners, their teachers, their parents and the principal. Data collection from the sampled participants continued until data saturation was reached (Wolcott 2009:45) on the patterns of aggression. According to Agar (1998:20) data saturation refers to the repeating of information during observation and interviews.

### Data collection

According to Wolcott (2009:41–44) one of the ‘unsung features’ of ethnographic research is its embracing of multiple data collection techniques. The fieldwork was conducted by means of prolonged engagement with the Grade 10 learners in the secondary school by one of the researchers. The focus was on participant observation in order to identify the ‘rich points’ in exploring and describing the culture of aggression. Artefacts and other school documents, like minutes of meetings of teachers and the school-based-support team, the school's website and Facebook page, were also taken into consideration (Denzin & Lincoln 2006:103). Observations were made, graffiti studied, language noted, documents scrutinised, reports analysed, and even informal talks held with involved persons such as the learners, teachers and members of the school governing body. Field notes were made consistently to support observations by the researcher conducting the fieldwork.

The meaning of the identified rich points pertaining to a culture of aggression was further explored by conducting interviews with learners, parents, the principal and teachers. Individual in-depth semi-structured face-to-face interviews were used to explore the phenomenon in question as an open conversation on a specific topic (Service 2010:4–5). The interviews (Table 1) assisted us in understanding the culture of aggression from the individuals’ perspective. The interviewing researcher created an atmosphere in which individuals narrated their stories according to their own perceptions (Holloway & Wheeler 2010:87-88). The interviews were tape-recorded and transcribed verbatim.

The multi-data collection ensured that this ethnography worked towards providing a qualitative, insightful and triangulated description of the patterns of a culture of aggression amongst learners in the Grade 10 A class.

**TABLE 1:** Participants and the interviews and conversations that were conducted.

Participants	Number of interviews and conversations
Learners from Grade 10 A	15
Parents of learners from Grade 10 A	7
Teachers from learners of Grade 10 A	10
School management team	8
School governing body	8

### Data analysis

According to Bonta (2009:142) data analysis should assist researchers in identifying the threads that can be woven together to tell a coherent, focused and comprehensible story of the social life that was observed and recorded. It should permit ethnographers to discover patterns in data and link them together into comprehensive descriptions. The data of this research were used to describe the culture of aggression prevalent amongst learners in the Grade 10 A class. The data were analysed by applying Tesch's approach (Creswell 2007:156–157).

Excerpts from clarified observations and verbatim quotations from the transcribed individual interviews with learners, parents, teachers, school management team and school governing body were interwoven in this account. This was used to convey the participants’ understanding of their day-to-day encounters in the school as an organisation (Neuman 2000:149). Expression was given by using their own symbols, language, concepts and subjective theories (Holloway & Wheeler 2010:6–7). A clean set of data, including field notes, interviews and documents, was given to an independent coder with experience in qualitative data analysis, who analysed the data independently. The researchers then had a consensus discussion with the independent coder on the findings of the data analysis.

## Ethical considerations

Ethical principles were complied with, to safeguard the dignity, rights, safety and well-being of all the participants in this study (Burns & Grove 2009:209). Dhai and McQuiod-Mason (2011:40) draw attention to the importance of ethnographers’ enactment of an ethical code, because they do not work in a vacuum, they work with people. Participants’ innermost, sacred rites, achievements and failures are researched. In pursuing this, we subscribed to a code of ethics that preserved the participants’ rights, facilitated communication in the field, and left the door open for further research. Therefore we engaged the participants in a principled pursuit with a sense of professional responsibility at all times (Dhai & McQuiod-Mason 2011:41–42).

Formal consent was obtained from the Gauteng DoE followed by obtaining approval from the Ethics Committee of the Faculty of Education of the responsible university. Thereafter consent was given by the school principal and the parents of the participants. Lastly, the participants gave their personal consent (see also the South African Constitution [Republic of South Africa 1996], *Act* no. 108 of 1996). Participants were made aware of their rights and that they were participating voluntarily in the research and could thus withdraw at any time should they chose to do so. After completion of the research interviews, possible misconceptions that might have arisen in the minds of participants were rectified by reflecting with them on the interactions.

### Trustworthiness

Guba's model of trustworthiness (De Vos, Schurink & Strydom 2011:419–421) was applied to ensure trustworthiness. The four criteria for trustworthiness are truth value through the strategy of credibility, applicability through the strategy of transferability, consistency through dependability, and neutrality through confirmability. To ensure credibility one of the researchers spent more than 12 months in the field observing the participants in their context before the interviewing started. In addition, triangulation was done by using different methods of data collection: multiple observations, interviews, documents, photographs, graffiti and audiovisual material. Discussions were also held with relevant peers, colleagues and participants. Transferability was ensured by providing the demographics of the participants and a rich description of the results, with supporting direct quotations from the interviews. Dependability was ensured by providing a dense description of research methodology. Finally, confirmability was ensured by providing a chain of evidence throughout the research process by means of a confirmability audit.

## Discussion of results

Multi-data collection provided a rich description of the patterns of aggression in a secondary school. It became evident during the researchers’ fieldwork that the secondary school under study was situated in an area that is extremely congested, with informal settlements and a high population density. A congested informal settlement was seemingly often the melting pot for a culture of aggression, which was mirrored by the learners in this Grade 10 A class in their school environment. A large number of taverns in the school's vicinity also seemed to encourage substance abuse. The school's doors and windows were broken, the school fence was relatively low and it had holes, which allowed easy and free access to anyone. The school walls were covered in graffiti.

### Patterns of a culture of aggression in a Grade 10 class

The findings outlined the patterns of a culture of aggression amongst learners as perceived by the Grade 10 learners, teachers, parents and principal. The findings are supported by descriptions of the physical environment of the class, the secondary school and the environmental context. Descriptions of the observations and interviews are evidenced by field notes, reflections and verbatim quotations. A culture of aggression in this Grade 10 A class is described in terms of the following patterns: patterns of anger, patterns of bullying, patterns of fighting, and patterns of challenges to moral values.

#### Patterns of anger

The patterns of aggression in the Grade 10 A class were defined by the external expression of anger that was observed in facial expressions, body language, physiological responses and, at times, public acts of aggression. Learners were angry with peers, teachers, parents and the schooling system. Their anger was based on different issues like a lack of resources, lack of parental financial support, physical needs not being met, psychological needs not being met, an unsafe school environment, rigid school rules, being bullied, not getting their way and being humiliated by teachers. The following are excerpts from the participants’ interviews:

‘Angry learners are more aggressive than their peers. Angry learners are on the increase lately, on daily basis angry learners are seen banging the classroom doors and chatting back to the teachers.’ (Teacher #2)

‘Anger gives birth to aggression and anger does play a role in sustaining aggressive behaviours, angry learners always do bad things to hurt others.’ (School Governing Body #8)

‘Learners are full of anger; sometimes I wonder what can one do. We are powerless under these calamities.’ (Teacher #5)

‘As for me, I do not worry about a lot of things, I choose not to get involved, and just stay out of trouble or just pretend as if things are not happening to me, you know, like name calling, I don’t care because they call me names like bookworm … nerd … geek and so.’ (Learner #15)

Initially researchers thought that being unable to achieve some goals increases people's readiness to behave aggressively. This led to the ‘frustration-aggression theory’ (Myers 2011:576; Sadock & Sadock 2007:150). Frustration creates anger, which may generate aggression. Social psychologists later realised that physical pain, personal insults and other unpleasant events also instigate aggression. They viewed frustrations as simply being instances of adverse events (Myers 2011:578).

#### Patterns of bullying

The Grade 10 learners who were bullied in the classroom or in the secondary school environment did not see the school as a safe or healthy place. The researchers observed the patterns of bullying manifesting in different forms. There was bullying by social class, bullying by status, bullying by revenge and bullying for affiliation. Bullying by social class was common amongst the poor and middle- class learners, and was extremely noticeable amongst girls. Bullying by status was perpetuated by the popularity of a particular dominating gang that bullies the up-and-coming gangs. Bullying by revenge was created by a vicious circle of bullying. The learners who were bullied earlier in life tended to bully others as time progressed. Bullying for affiliation always took place when the representatives of different gangs canvassed for new members to join their gangs. The following are excerpts from the participants’ interviews:

‘I stay at home at least twice a week because I’m being bullied. I am afraid of them and I do not want to show that I am scared. Sometimes I am afraid that I will be suspended from school if I am caught involving myself with the bullies or retaliating.’ (Learner #8)

‘Most of the time I didn’t know what to do. I always fear what may happen next because I’m just standing this. Sometimes I fear what can happen next; I’m so scared that I will lose it. What if I do?’ (Parent #6)

There is considerable evidence that psychological disorders, low self-esteem, low self-confidence and poor social skills are amongst the many problems developed by learners who are bullied (Hamilton & Reati 2010). The effects of bullying are often demonstrated through poor academic achievement, a lack of engagement in class, disruptive behaviour and antagonistic peer relationships (Hamilton & Reati 2010).

#### Patterns of fighting

Oppositional and defiant fighting, temper tantrums and antagonistic behaviour form the patterns of fighting that were observed amongst the Grade 10 learners. The learners were being oppositional and defiant towards everything in the school. Learners seemed to always be fighting, sometimes without even knowing the reasons for their fighting. The more the teachers intervened in order to resolve the learners’ fights, the more the learners acted defiantly.

It was common for the learners to start their fights by being verbally abusive. That was how they struck out at each other's nerves. The antagonistic behaviour between the learners sometimes occurred when the learners became angry at being provoked by peers, and retaliated by hitting them. Sometimes two or more learners were engaged in an argument that escalated into an exchange of punches. Some of the learners acted fast in exchanging punches, as they failed to manage their anger effectively. The differences in fights only depended on the nature and the cause.

Fights were frequent amongst both genders, although it was observed that the fights amongst the Grade 10 learners differed according to gender. Sometimes the girls were engaged in a heated conversation which led to a fight, and at other times the fights were one-on-one or there were group fights involving only girls. The fights were rarely a mixture of girls and boys. Commonly the boys were engaged in their boys-only fights that included weapons and fewer insults. The following are excerpts from the participants’ interviews:

‘Girls tend to bicker and cry and tattle. Boys tend to be more aggressive and physically violent.’ (School management team #3)

‘Boys are a lot more physical and girls use their mouths.’ (School management team #3)

‘Our number of aggressive girls is increasing. In general aggression rates are definitely going up. Passive aggression is also going up. It used to be boys who were more aggressive, but not any more. Yesterday a couple of girls instigated a fight on the playfield. The girls and the boys were all fighting physically.’ (School management team #1)

Fighting in schools is described as behaviour that seriously disrupts a safe learning environment in a classroom or school (MacDonald, Gilmer & Collings 2006:83).

### Patterns of challenges to moral values in education

Moral values in education should reflect the common rights (Merriam Webster Dictionary Company 2014) that learners should strive for in a secondary school. Common rights include a right to experience a safe environment, a right not to be bullied and a right to a preferred sexual orientation. A lack of the upholding of moral values in education was present amongst the Grade 10 learners. Learners were frequently disrespectful of the needs of individuals, they did not understand nor tolerate differences in personality, and other learners’ uniqueness was neither accepted nor tolerated in this Grade 10 class.

The learners were coerced by peers into forming what they termed a ’uniform identity‘. For example, if the norm was to cause chaos in the classroom, all the learners were to follow suit. If not, those learners who maintained order were regarded as outsiders. They were labelled as ’misfits‘, perceived to be losers and subjected to ridicule. The following are excerpts from the participants’ interviews:

‘With the school environment being a virtually police-free milieu, learners are able to smuggle alcohol and drugs into the school premises. In this case, learners were exposed to alcohol and drugs safe from the hands of the law. Substance abuse will be indulged with mostly in the unsuspecting attention of the teachers.’ (School management team #6)

‘It's very stressful … Very. You feel like there is no purpose in coming to school anymore. I feel so bad because we do not feel safe and secure in the classroom. It makes me feel like quitting this school. Maybe other schools in other places respect the school laws.’ (Teacher #10)

Buie (2004:139) states that often victims of aggression can in later situations become the perpetrators of aggression. Thus they then accede to what at first they viewed as not acceptable. It can happen that they rationalise that aggression is indeed a fun-filled payback. They seem to ignore reflecting on their own aggression under such circumstances, putting their own mental health at risk.

## Limitations

The findings are contextual and cannot be generalised to other classrooms in secondary schools in the Sedibeng District.

## Conclusion and recommendations

The overall picture and implications that result from the analysis of observations and quotations demand intervention. Prevalent in the Grade 10 A class were patterns of anger, bullying, physical fighting and ignorance regarding the human rights of individual learners which underpin moral values. The culture of aggression amongst learners poses a challenge to the school, the principal, the management of the school and the parents.

Viewed in perspective, the learners who were fanning the flames of the destructive culture of aggression were simultaneously also the victims of a self-inflicted culture of aggression. A cycle and spiral of aggression resulting in destruction of intra- and interpersonal relationships was present. At the root of the studied culture of aggression were the neglect of and non-adherence to human rights and a sound base of morals. It is almost as if a survival-of-the-fittest scenario was prevalent. This happened in a context of poverty, high population density and low educational outcomes, in spite of high expectations from the community. Basic resources were scarce and often non-existing. Urbanisation and disruption of traditional values were prominent. Counteracting these patterns of a culture of aggression is a challenging task.

In addressing these challenges intrapersonal respect and self-love must be facilitated in a context where food and obtaining other basic needs are often more desirable. The learners should be assisted in trying to see the bigger picture. Victor Frankl (1985) claims that one might not be able to do anything to one's circumstances and accompanying challenges, but how these challenges are viewed is often the only way towards a better future. Nevertheless, one cannot ignore the reality of the here and now in which these children are entrapped.

These learners should thus be assisted to value themselves as individuals with a future. The present is important in realising a possible future and making it a reality in spite of challenges. To facilitate this, values and morals are necessary. To become aware and have self-respect are necessary; to treat oneself with sensitivity can also not be ignored.

Once intrapersonal awareness is kindled, interpersonal respect should be facilitated (Kneisl, Wilson & Trigoboff 2004:293–299). This is important because when disrespect for interpersonal issues and the upholding of a culture of aggression are fanned, this might lead to intrapersonal challenges. Patterns of acting in a disrespectful manner towards other learners by showing anger, bullying and physically attacking them do not facilitate self-respect and an individual's own mental health. Under such circumstances sensitivity to the needs and challenges of classmates cannot be cultivated.

To address the patterns of disrespect, learners should be assisted to act with sensitivity (Kneisl et al. 2004:293–299) towards other learners and to evade acting intrusively towards other learners. Resilience should be cultivated in learners. In addition, an understanding should be fostered of the outcomes of disrespectful actions and verbalisation, as well as acts of insensitivity towards fellow classmates.

Clark (2003:468) asserts that on many occasions interventions to constructively manage a culture of aggression involve advocacy, which may take place with the individual learner or group of learners. The constructive management of a culture of aggression should encompass school safety and security issues in the school environment, together with procedures that intend to deal with the matters and consequences of breaching safety and security ­– for example, safety rules and procedures that deal with matters such as late-coming, criminal acts and conducting searches and seizes (Bear 2009:14). According to Loo (2008:120) feeling safe in the school environment extends beyond the individual; it is the perception that incorporates learners’ mental health when they are present in the classroom.

The present day calls for a paradigm shift: the security personnel should understand accountability, verbal and non-verbal aggression, use of technology like cell phones and computers in aggression, substance abuse, a variety of weapons and electronics (Harvey 2011:5). Some educational institutions began to respond to safety demands with carefully crafted security programmes that address a variety of educational and security topics, and which incorporate parents (Harvey 2011:5).

In conclusion, the culture of aggression amongst the learners in a Grade 10 class was their reality – a daily challenge. These learners promoted and experienced a culture of aggression. The challenge is to assist the involved learners to respect each other's human dignity. In cultivating such an approach towards each other, relationships can be developed in which those involved would be acting with sensitivity towards each other's needs. Such relationships often also result in the development of self-respect and a nuanced future orientation (Kneisl et al. 2004:293–299) as part and parcel of mental health.
